# Association Between Treatment-Emergent Pancreatic Volume Loss and Survival Outcomes in Patients with Metastatic Colorectal Cancer Receiving Bevacizumab

**DOI:** 10.3390/medicina62081459

**Published:** 2026-07-28

**Authors:** Hatice Ayyıldız Sevim, Hayriye Şahinli, Galip Can Uyar, Selahattin Çelik, Salih Karatlı, Güner Akgüner, Berna Turhan, Sümeyya Duran Kaymak, Rasime Pelin Kavak, Gökşen İnanç İmamoğlu

**Affiliations:** 1 Department of Medical Oncology, Ankara Etlik City Hospital, 06170 Ankara, Türkiye; dr.hayriye@hotmail.com (H.Ş.); g.can_uyar@hotmail.com (G.C.U.); drcelikselahattin@gmail.com (S.Ç.); karatlisalih@hotmail.com (S.K.); gunerakguner@gmail.com (G.A.);; 2 Department of Radiology, Ankara Etlik City Hospital, 06170 Ankara, Türkiye; 3 Department of Radiology, Lokman Hekim University Akay Hospital, 06700 Ankara, Türkiye

**Keywords:** metastatic colorectal cancer, bevacizumab, pancreatic volume loss, pancreatic atrophy, survival

## Abstract

*Background and Objectives*: The effect of bevacizumab on the morphology of non-tumoral organs remains unclear. We evaluated computed tomography (CT)- derived treatment-emergent pancreatic volume loss and examined its association with progression-free survival (PFS) and overall survival (OS) in patients with metastatic colorectal cancer receiving bevacizumab. *Materials and Methods*: This single-center retrospective study included 105 patients treated at Ankara Etlik City Hospital who had evaluable paired contrast-enhanced CT scans obtained before and during therapy. Pancreatic volume was measured on CT, and percentage volume loss was calculated. Clinical, laboratory, radiological, and survival data were retrospectively reviewed. *Results*: Mean pancreatic volume declined from 89.9 ± 6.1 mL before treatment to 83.6 ± 5.1 mL during therapy (*p* < 0.001). Albumin levels and platelet counts also decreased, whereas amylase, hemoglobin, and the neutrophil-to-lymphocyte ratio remained largely unchanged. In the main multivariable models, each 1% increase in pancreatic volume loss was associated with worse bevacizumab-line PFS (HR 1.06, 95% CI 1.02–1.10; *p* = 0.032) and OS (HR 1.08, 95% CI 1.01–1.15; *p* = 0.041). Percentage change in albumin was independently associated with both endpoints. Sensitivity analyses supported a more consistent association of pancreatic volume loss with PFS than with OS. *Conclusions*: Pancreatic volume decreased during bevacizumab-containing therapy. Greater pancreatic volume loss was associated with shorter PFS and OS; however, the association was more consistent for PFS. Prospective studies are needed to validate these findings and determine their clinical relevance.

## 1. Introduction

Colorectal cancer accounts for a substantial proportion of the global cancer burden. Based on GLOBOCAN 2022 estimates, approximately 1.9 million individuals were diagnosed with colorectal cancer worldwide, and nearly 904,000 deaths were attributed to the disease, placing colorectal cancer third in incidence and second in cancer-related mortality [1]. Approximately 20–30% of patients with colorectal cancer present with metastatic disease at diagnosis [2], while metachronous distant metastases may also develop during follow-up in patients who undergo curative surgery. Despite advances in current treatment strategies, the reported rates of metachronous metastasis after curative surgery range from approximately 10% to 25% [3,4].

Systemic chemotherapy remains the basis of current treatment for unresectable metastatic or recurrent colorectal cancer [2]. When doublet or triplet chemotherapy regimens are combined with targeted therapies, including antiangiogenic agents, response rates in metastatic colorectal cancer can reach 60–70%, with corresponding improvements in survival outcomes [5,6]. Bevacizumab, a monoclonal antibody targeting vascular endothelial growth factor (VEGF), is widely used as part of both first- and second-line treatment strategies for metastatic colorectal cancer [7]. Its antitumor activity is mainly mediated through suppression of VEGF-driven angiogenesis, thereby reducing tumor vascular formation.

However, the effects of VEGF inhibition may not be limited to tumor tissue, as prolonged antiangiogenic exposure may also induce structural alterations in non-malignant tissues. Previous studies have linked bevacizumab exposure to reduced brain volume in patients with high-grade gliomas and to skeletal muscle depletion in metastatic colorectal cancer [8,9]. With regard to the pancreas, the CT-based analysis by Phillip et al. showed a significant reduction in pancreatic volume among patients receiving bevacizumab-containing therapy, whereas no comparable change was observed in the chemotherapy-only control group [10]. Similarly, another study in patients with colorectal cancer reported significant reductions in both hepatic and pancreatic volumes during bevacizumab-based chemotherapy, suggesting possible antiangiogenic therapy-related organ atrophy [11].

Pancreatic volume reduction during bevacizumab-containing therapy has been previously reported; however, the clinical significance of this finding remains unclear. In particular, the association between treatment-emergent pancreatic volume loss and survival outcomes has not been well established. Therefore, this study evaluated computed tomography (CT)-derived pancreatic volume loss during bevacizumab-containing therapy and examined its association with progression-free survival (PFS) and overall survival (OS) in patients with metastatic colorectal cancer receiving bevacizumab.

## 2. Materials and Methods

### 2.1. Study Design and Patients

This study was designed as a single-center, retrospective observational study conducted at Ankara Etlik City Hospital. Medical records of patients with colorectal cancer followed between December 2022 and June 2025 were reviewed retrospectively. Patients were eligible if they had metastatic colorectal cancer, received bevacizumab-based systemic therapy, had no known diagnosis of diabetes mellitus, and had evaluable paired contrast-enhanced CT images obtained before and after treatment.

Patients were excluded if they had known diabetes mellitus or a history of clinically significant alcohol use, a concurrent or previous second primary malignancy, a history of pancreatic surgery, acute or chronic pancreatitis, or documented pre-existing pancreatic atrophy, unavailable or inadequate paired pre- and post-treatment contrast-enhanced CT images, or missing key clinical, laboratory, or follow-up data.

The study protocol was approved by the Ankara Etlik City Hospital Scientific Research Evaluation and Ethics Committee (AEŞH-BADEK2-2025-249, 8 July 2025). The study was conducted in accordance with the principles of the Declaration of Helsinki. Due to the retrospective design of the study and the use of anonymized patient data, the requirement for informed consent was waived.

### 2.2. Pancreatic Volume Assessment

Pancreatic volume was evaluated using paired contrast-enhanced abdominal CT images obtained before and after bevacizumab-based therapy. The pre-treatment CT scan was defined as the most recent contrast-enhanced CT examination performed before the initiation of bevacizumab. The post-treatment CT scan was defined as the follow-up contrast-enhanced CT examination obtained at least 12 weeks after the initiation of bevacizumab-based therapy. In patients who continued bevacizumab for 24 weeks or longer, the CT scan closest to the 24-week time point was preferentially used for post-treatment volumetric assessment.

Pancreatic volume was manually measured on axial CT images using the FONET Picture Archiving and Communication System (PACS) (FONET Bilgi Teknolojileri A.Ş., Ankara, Türkiye) by delineating the pancreatic parenchyma from the head to the tail on consecutive slices. Adjacent vascular structures, bowel loops, peripancreatic fat, and non-pancreatic tissues were excluded from the measurement area. Pancreatic volume was recorded in milliliters (mL). Percentage pancreatic volume loss was calculated as follows: [(pre-treatment pancreatic volume − post-treatment pancreatic volume)/pre-treatment pancreatic volume] × 100. Higher values indicated greater pancreatic volume reduction during treatment.

### 2.3. Clinical and Laboratory Variables

Patient-level data were retrospectively reviewed using the hospital electronic record system. Demographic characteristics, clinical and pathological information, molecular findings, treatment details, and laboratory results were included in the assessment. The main variables analyzed were age, sex, body mass index, Eastern Cooperative Oncology Group (ECOG) performance status, primary tumor site, stage at initial diagnosis, metastatic sites and their number, RAS/BRAF mutation status, bevacizumab treatment line, concomitant chemotherapy regimen, and total number of bevacizumab cycles. Patients were referred to a dietitian for nutritional assessment when clinically indicated as part of the multidisciplinary evaluation.

Laboratory parameters were recorded before and after bevacizumab-based therapy in accordance with the timing of radiological assessment. The evaluated laboratory parameters included hemoglobin, albumin, amylase, neutrophil count, lymphocyte count, and platelet count. The neutrophil-to-lymphocyte ratio (NLR) was calculated by dividing the absolute neutrophil count by the absolute lymphocyte count.

Percentage changes in NLR, albumin, amylase, and platelet count were calculated using the following formula: [(post-treatment value − pre-treatment value)/pre-treatment value] × 100. Percentage pancreatic volume loss was calculated separately as [(pre-treatment pancreatic volume − post-treatment pancreatic volume)/pre-treatment pancreatic volume] × 100.

### 2.4. Statistical Analysis

Continuous variables were summarized as mean ± standard deviation (SD) or median with interquartile range (IQR), according to distributional characteristics. Categorical variables were presented as frequencies and percentages. The distribution of continuous variables was assessed using visual inspection of histograms and Q–Q plots, together with normality testing when appropriate.

Pre-treatment and post-treatment pancreatic volume and laboratory parameters were compared using the paired samples *t*-test for normally distributed variables and the Wilcoxon signed-rank test for non-normally distributed variables.

The relationship between percentage pancreatic volume loss and continuous clinical or laboratory variables was examined with Spearman correlation. Univariable and multivariable linear regression models were then constructed to explore variables potentially related to pancreatic volume loss. These models included age, body mass index (BMI), number of bevacizumab cycles, and percentage changes in neutrophil-to-lymphocyte ratio (NLR), albumin, amylase, and platelet count.

Kaplan–Meier analysis was used for survival assessment. Bevacizumab-line progression-free survival (PFS) was calculated from the date of bevacizumab-containing treatment initiation to radiological or clinical progression or death from any cause. The first of these events was accepted as the PFS endpoint. Bevacizumab-line overall survival (OS) was measured from the same treatment start date to death from any cause. Patients without progression or death at the last follow-up were censored in the relevant analyses. Median survival estimates and 95% confidence intervals (CIs) were derived from Kaplan–Meier curves. OS from the date of diagnosis and first-line PFS were also reported descriptively.

Variables potentially influencing bevacizumab-line PFS and OS were examined using univariable and multivariable Cox proportional hazards models. Effect estimates were expressed as hazard ratios (HRs) with 95% confidence intervals (CIs). Variables with a *p*-value < 0.10 in univariable analysis, together with clinically relevant covariates, were selected as candidates for the multivariable models. For continuous variables, HRs were interpreted per 1-unit increase, whereas for percentage-change parameters, HRs reflected the effect of each 1% increase. Chemotherapy backbone was grouped as irinotecan-containing versus non-irinotecan-containing regimens, with the latter used as the reference category. To examine the consistency of the findings and reduce potential heterogeneity related to bevacizumab treatment line, sensitivity analyses were conducted in the subgroup of patients who received bevacizumab as first-line therapy.

As pancreatic volume loss and laboratory changes were measured during treatment, fixed 3-month landmark sensitivity analyses were applied to limit time-dependent measurement bias. Patients who had an event or were censored before month 3 were not included in the corresponding landmark analysis. In these analyses, survival time was redefined to start at the 3-month landmark, and Cox proportional hazards models for bevacizumab-line PFS and OS were reconstructed in the landmark-eligible population.

Restricted cubic spline models with three knots were used to assess whether the association between pancreatic volume loss and bevacizumab-line survival outcomes followed a non-linear pattern. The median pancreatic volume loss of the cohort was selected as the reference point. The proportional hazards assumption in Cox models was checked using Schoenfeld residuals. Collinearity among variables entered into the multivariable models was evaluated with variance inflation factor (VIF) values, all of which were <5.

Because no missing values were present for the variables included in the primary analyses, the analyses were conducted on the complete dataset. Before statistical testing, the dataset was checked for outliers and potential inconsistencies; errors confirmed to result from data entry were corrected using the original source records. All statistical procedures were carried out with IBM SPSS Statistics version 25.0 (IBM Corp., Armonk, NY, USA) and Python version 3.12. Statistical significance was defined as a two-sided *p*-value < 0.05.

## 3. Results

**Figure 1:**
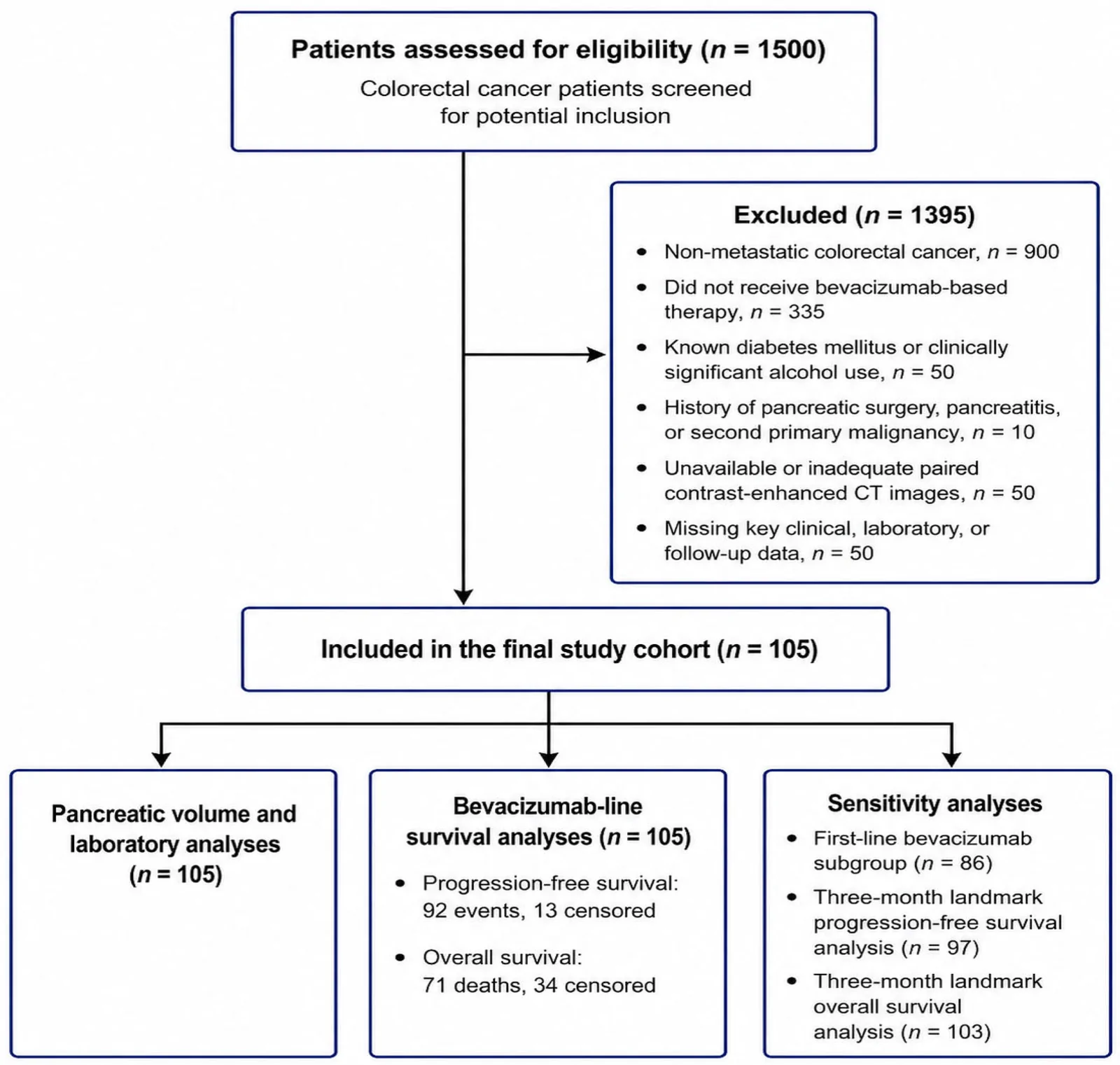
Study flow chart. Abbreviations: CT, computed tomography; OS, overall survival; PFS, progression-free survival.

During the study period, records of 1500 patients followed with a diagnosis of colorectal cancer were retrospectively reviewed. Patients without metastatic disease (*n* = 900) and those who had not received bevacizumab-containing therapy (*n* = 335) were excluded. Among 265 patients treated with bevacizumab-containing regimens, 160 were considered ineligible because of known diabetes mellitus or clinically significant alcohol use, a history of pancreatic surgery, pancreatitis, or second primary malignancy, lack of adequate paired contrast-enhanced CT images, or incomplete key clinical, laboratory, or follow-up data. After this screening process, 105 patients constituted the final analysis cohort. The patient selection flow is presented in Figure 1.

In the final cohort, the median age was 68.6 years (IQR: 58.3–74.1), and 68 patients (64.8%) were aged 65 years or older. There were 46 male (43.8%) and 59 female patients (56.2%). The primary tumor was located in the right colon in 40 patients (38.1%), the left colon in 38 patients (36.2%), and the rectum in 27 patients (25.7%). At initial diagnosis, 5 patients (4.8%) had stage II disease, 14 (13.3%) had stage III disease, and 86 (81.9%) had stage IV disease.

Regarding metastatic disease distribution, liver involvement was observed in 76 patients (72.4%), lung involvement in 26 patients (24.8%), peritoneal involvement in 24 patients (22.9%), lymph node involvement in 25 patients (23.8%), bone involvement in 6 patients (5.7%), and ovarian involvement in 3 patients (2.9%). A single metastatic site was present in 59 patients (56.2%), whereas 46 patients (43.8%) had two or more metastatic sites. Bevacizumab was used in the first-line setting in 86 patients (81.9%) and in the second-line setting in 19 patients (18.1%). Treatment regimens consisted of FOLFOX plus bevacizumab in 72 patients (68.6%), FOLFIRI plus bevacizumab in 30 patients (28.6%), and capecitabine plus bevacizumab in 3 patients (2.9%). The median number of bevacizumab cycles was 12 (IQR: 7–20). Baseline clinical, molecular, treatment-related, and laboratory characteristics are presented in Table 1.

A significant reduction in pancreatic volume was observed after bevacizumab-based therapy. The mean pancreatic volume was 89.9 ± 6.1 mL before treatment and decreased to 83.6 ± 5.1 mL after treatment (*p* < 0.001). Among laboratory parameters, albumin level and platelet count also showed significant declines. Albumin decreased from 38.8 ± 4.3 g/L to 35.7 ± 6.6 g/L, while platelet count decreased from 359.7 ± 122.0 × 10^9^/L to 237.0 ± 106.8 × 10^9^/L (*p* < 0.001 for both). No statistically significant differences were detected in amylase, hemoglobin, or NLR between the pre-treatment and post-treatment measurements. Changes in pancreatic volume and laboratory parameters during treatment are summarized in Table 2.

**Figure 2:**
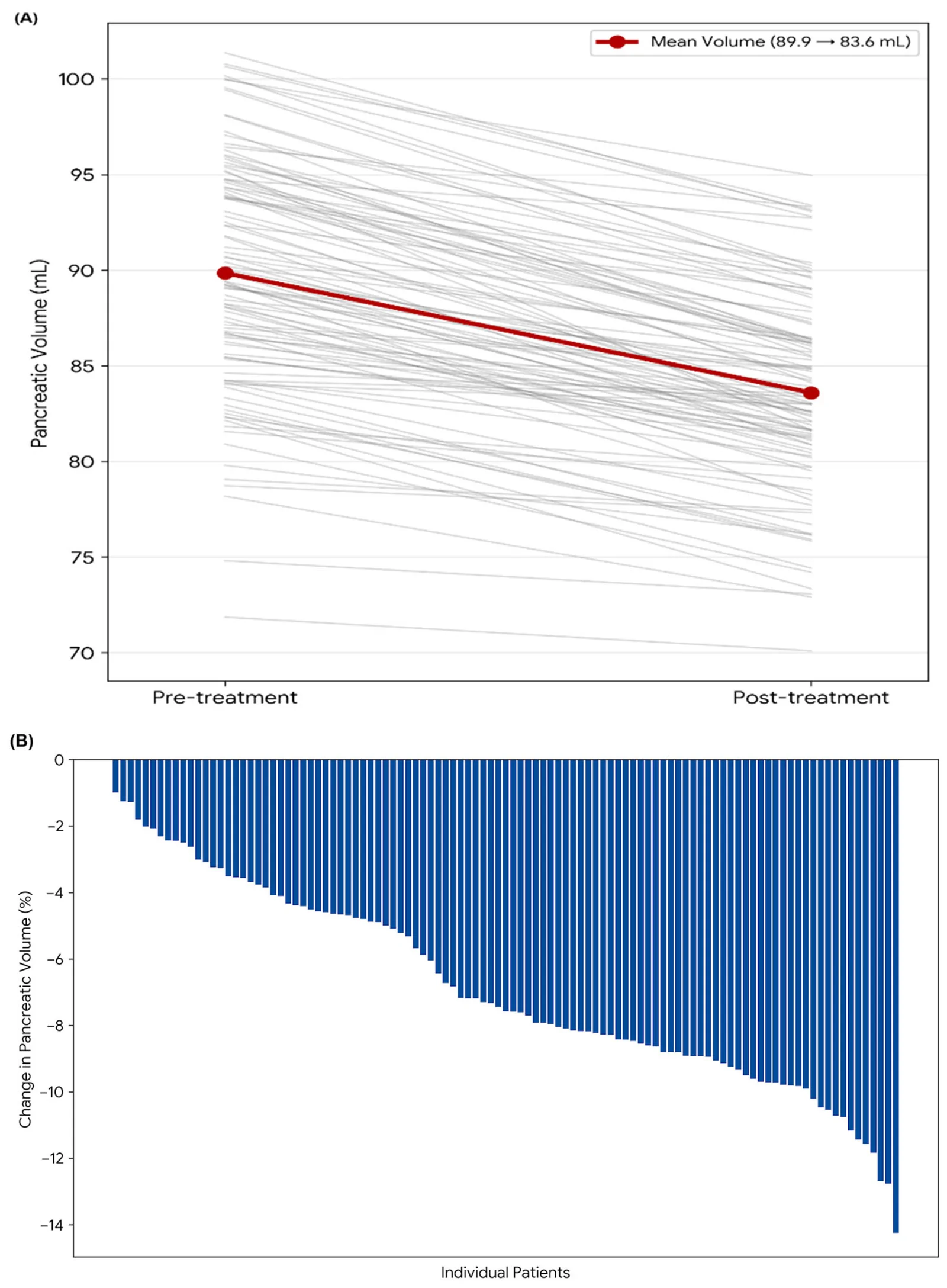
Morphological changes in the pancreas during bevacizumab-based therapy. (**A**) Paired line plot showing individual (gray lines) and mean (red line) changes in pancreatic volume (mL) from pre-treatment to post-treatment. (**B**) Waterfall plot depicting the percentage change in pancreatic volume for each individual patient (*N* = 105). Abbreviations: mL, milliliters.

Morphological changes in pancreatic volume are illustrated in Figure 2. In the paired patient-level assessment, most patients showed a reduction in pancreatic volume after treatment. The mean pancreatic volume decreased from 89.9 ± 6.1 mL before treatment to 83.6 ± 5.1 mL after bevacizumab-based therapy (*p* < 0.001). The waterfall plot demonstrated interpatient variability in the percentage change in pancreatic volume, while the overall pattern indicated a predominant tendency toward volume loss.

Percentage change in amylase showed a weak inverse association with pancreatic volume loss (r = −0.199, *p* = 0.042). No significant correlations were identified for the other evaluated variables (Table 3).

In the linear regression analyses, none of the evaluated variables showed a statistically significant association with pancreatic volume loss. Age, BMI, number of bevacizumab cycles, ΔNLR, Δalbumin, Δamylase, and Δplatelet count were not significant in either the univariable or multivariable models (Table 4).

In the bevacizumab-line PFS analysis, 92 progression or death events were recorded. In univariable Cox analysis, ECOG ≥ 2, pancreatic volume loss, ΔNLR, Δalbumin, Δamylase, and Δplatelet count were identified as variables related to PFS. In the multivariable model, ECOG ≥ 2 maintained its adverse association with PFS (HR: 1.78, 95% CI: 1.06–2.99, *p* = 0.028). The presence of peritoneal metastasis was also independently linked to a greater risk of progression (HR: 2.06, 95% CI: 1.25–3.39, *p* = 0.005). Each 1% increment in pancreatic volume loss corresponded to an increased progression risk (HR: 1.06, 95% CI: 1.02–1.10, *p* = 0.032). Percentage change in albumin also remained significant in the model (HR: 0.99, 95% CI: 0.97–1.00, *p* = 0.015). By contrast, ΔNLR, Δamylase, and Δplatelet count did not show independent significance in the multivariable analysis (Table 5).

In the bevacizumab-line OS analysis, 71 death events were evaluated. In univariable analysis, ECOG ≥ 2, peritoneal metastasis, BRAF mutation, pancreatic volume loss, ΔNLR, Δalbumin, and Δamylase were identified as variables related to OS. In the multivariable model, ECOG ≥ 2 was linked to a markedly higher mortality risk (HR: 2.98, 95% CI: 1.71–5.22, *p* < 0.001). Peritoneal metastasis also retained independent significance for OS (HR: 2.60, 95% CI: 1.49–4.55, *p* < 0.001). Each 1% increment in pancreatic volume loss corresponded to an increased risk of death (HR: 1.08, 95% CI: 1.01–1.15, *p* = 0.041). Percentage change in albumin also remained significant in the multivariable model (HR: 0.98, 95% CI: 0.96–0.99, *p* = 0.002). By contrast, BRAF mutation, ΔNLR, and Δamylase did not show independent significance in the multivariable analysis (Table 6).

In the overall cohort of 105 patients, median bevacizumab-line PFS was 8.84 months (95% CI: 6.77–11.04). Using the same treatment-line starting point, median OS was 14.23 months (95% CI: 10.87–18.79). When survival was assessed from the date of diagnosis, median OS was 20.86 months (95% CI: 17.15–25.72), while median PFS from the start of first-line systemic therapy was 11.93 months (95% CI: 10.45–12.81).

In the subgroup of 86 patients who received bevacizumab in the first-line setting, the median PFS calculated from the start of bevacizumab-based therapy was 8.84 months (95% CI: 6.80–11.37), and the median OS was 15.97 months (95% CI: 10.87–19.45). In this subgroup, the median OS from diagnosis was 19.42 months (95% CI: 14.62–29.17), and the median first-line PFS was 11.99 months (95% CI: 10.45–13.60). Detailed survival estimates are provided in Supplementary Table S1.

In the subgroup of patients who received bevacizumab as first-line therapy, 75 PFS events were recorded. In univariable analysis, ECOG ≥ 2, pancreatic volume loss, ΔNLR, Δalbumin, Δamylase, and Δplatelet count were identified as variables related to PFS. In the multivariable model, pancreatic volume loss maintained independent significance; each 1% increase in volume loss corresponded to a higher risk of progression (HR: 1.04, 95% CI: 1.01–1.07, *p* = 0.043). Percentage change in albumin was another variable that remained significant in the model (HR: 0.985, 95% CI: 0.971–0.998, *p* = 0.029). By contrast, ECOG ≥ 2, liver metastasis, ΔNLR, Δamylase, and Δplatelet count did not show independent significance in the multivariable analysis (Supplementary Table S2).

In the first-line bevacizumab subgroup, 57 death events were evaluated in the OS sensitivity analysis. In univariable analysis, ECOG ≥ 2, BRAF mutation, pancreatic volume loss, ΔNLR, Δalbumin, and Δamylase were identified as variables related to OS. In the multivariable model, ECOG ≥ 2 was associated with a marked increase in mortality risk (HR: 3.31, 95% CI: 1.78–6.18, *p* < 0.001). Peritoneal metastasis (HR: 2.43, 95% CI: 1.31–4.52, *p* = 0.005) and BRAF mutation (HR: 4.39, 95% CI: 1.80–10.70, *p* = 0.001) also showed independent significance for OS. Percentage change in albumin was another variable that remained significant in the model (HR: 0.975, 95% CI: 0.959–0.990, *p* = 0.002). By contrast, pancreatic volume loss, ΔNLR, and Δamylase did not show independent significance in the multivariable analysis (Supplementary Table S3).

The 3-month landmark PFS analysis included 97 patients, among whom 84 PFS events were recorded. In univariable analysis, ECOG ≥ 2, liver metastasis, pancreatic volume loss, ΔNLR, and Δamylase were identified as variables related to PFS. In the multivariable model, pancreatic volume loss maintained independent significance; each 1% increase in volume loss corresponded to a higher risk of progression (HR: 1.08, 95% CI: 1.01–1.15, *p* = 0.047). ΔNLR (HR: 1.002, 95% CI: 1.001–1.004, *p* = 0.006) and Δamylase (HR: 0.995, 95% CI: 0.991–0.999, *p* = 0.011) also remained significant in the model. By contrast, ECOG ≥ 2 and liver metastasis did not show independent significance in the multivariable analysis (Supplementary Table S4).

The 3-month landmark OS analysis included 103 patients, among whom 69 death events were recorded. In univariable analysis, ECOG ≥ 2, peritoneal metastasis, BRAF mutation, pancreatic volume loss, ΔNLR, and Δamylase were identified as variables related to OS. In the multivariable model, ECOG ≥ 2 was associated with an increased mortality risk (HR: 2.56, 95% CI: 1.42–4.59, *p* = 0.002). Peritoneal metastasis (HR: 2.42, 95% CI: 1.37–4.28, *p* = 0.002) and BRAF mutation (HR: 2.70, 95% CI: 1.17–6.27, *p* = 0.021) also showed independent significance for OS. In addition, ΔNLR (HR: 1.002, 95% CI: 1.001–1.004, *p* = 0.005) and Δamylase (HR: 0.994, 95% CI: 0.989–0.998, *p* = 0.009) were other variables that remained significant in the model. By contrast, pancreatic volume loss did not emerge as an independent determinant of OS in the 3-month landmark multivariable analysis (Supplementary Table S5).

Restricted cubic spline modeling was used to evaluate whether the relationship between pancreatic volume loss and bevacizumab-line survival outcomes showed a non-linear pattern. In the PFS analysis, the estimated risk of progression tended to increase as pancreatic volume loss became greater (Supplementary Figure S1A). A similar upward trend was observed in the OS analysis, in which higher levels of pancreatic volume loss corresponded to an increased estimated risk of death (Supplementary Figure S1B). The reference point was defined as the median pancreatic volume loss of the cohort, which was 7.6%.

## 4. Discussion

Our findings demonstrated a marked reduction in pancreatic volume during bevacizumab-containing treatment in patients with metastatic colorectal cancer. Increasing pancreatic volume loss was accompanied by shorter bevacizumab-line PFS and OS in the main multivariable analyses. To our knowledge, this is the first study to examine the link between treatment-emergent pancreatic volume loss and survival outcomes in this patient population. Taken together, these findings support an association between treatment-emergent pancreatic volume loss and survival outcomes, particularly disease progression.

The observed reduction in pancreatic volume is consistent with previous CT-based studies suggesting that bevacizumab-containing treatment may be associated with non-tumoral organ volume loss. Phillip et al. reported a significant decrease in pancreatic volume from approximately 71.8 mL to 62.6 mL among patients treated with bevacizumab-containing regimens, whereas no comparable reduction was observed in patients receiving chemotherapy alone [10]. Notably, the observation that pancreatic volume reduction occurred in the bevacizumab-containing treatment group but not in the chemotherapy-only control group supports the possibility that pancreatic volume loss may be more closely related to bevacizumab-containing therapy than to cytotoxic chemotherapy alone. In the study by Oshiro et al., pancreatic volume decreased from 57.9 ± 16.0 mL to 47.4 ± 15.3 mL during bevacizumab-containing chemotherapy [11]. In the present study, pancreatic volume decreased from 89.9 ± 6.1 mL before treatment to 83.6 ± 5.1 mL after bevacizumab-based therapy. Although the magnitude of volume loss appeared smaller in our cohort, our findings support previous observations that bevacizumab-based therapy may be associated with measurable pancreatic volume reduction.

The molecular mechanisms underlying pancreatic atrophy in the human pancreas have not been fully elucidated. Pancreatic volume is known to be influenced by several factors, including pancreatic duct obstruction, chronic inflammation, age, growth factors, metabolic status, and rare genetic alterations [12,13,14,15,16]. Therefore, the pancreatic volume reduction observed during bevacizumab-based therapy is unlikely to be explained by a single mechanism. Nevertheless, previous studies have suggested that antiangiogenic therapies may lead to volume and functional loss in non-tumoral organs and that this process may be related to microangiopathic changes in normal tissues [17,18,19]. By inhibiting VEGF signaling, bevacizumab may reduce pancreatic microvascular circulation and contribute to gradual subclinical parenchymal atrophy.

In addition to pancreatic volume reduction, significant decreases in albumin level and platelet count were observed during bevacizumab-based therapy, whereas amylase, hemoglobin, and NLR did not change significantly. Although amylase levels did not change significantly during treatment, the percentage change in amylase showed only a weak inverse correlation with pancreatic volume loss. These findings suggest that the relationship between pancreatic volume reduction and biochemical pancreatic alteration may be limited, and that pancreatic volume loss may primarily represent a subclinical morphological change rather than overt pancreatic injury. The concomitant decrease in albumin may reflect systemic inflammation, nutritional deterioration, treatment exposure, and disease burden during the course of metastatic colorectal cancer. This interpretation is supported by recent studies showing that hypoalbuminemia and inflammation- or nutrition-based markers are associated with adverse clinical outcomes in colorectal cancer [20,21,22]. In the present study, albumin change was also associated with survival outcomes in multivariable analyses. In contrast, the decrease in platelet count may be more likely related to treatment exposure or chemotherapy-related myelosuppression and should be interpreted cautiously.

In the linear regression analyses, no significant association was found between pancreatic volume loss and the evaluated clinical or laboratory variables, including age, BMI, number of bevacizumab cycles, and percentage changes in NLR, albumin, amylase, and platelet count. The potential relationship between antiangiogenic treatment exposure and pancreatic atrophy has also been addressed in previous studies. Ganten et al. reported that pancreatic atrophy during sorafenib therapy was associated with cumulative dose and exposure time [18]. In contrast, Oshiro et al. showed that bevacizumab-associated pancreatic atrophy was not dependent on bevacizumab dose or exposure time. This difference may be related to the distinct pharmacologic mechanisms of sorafenib and bevacizumab. Sorafenib is a multikinase inhibitor targeting VEGF receptors and several other signaling pathways, whereas bevacizumab is a monoclonal antibody that specifically binds VEGF [11]. Therefore, pancreatic volume reduction during bevacizumab-based therapy may not necessarily show a simple dose- or duration-dependent pattern. Similarly, in our study, pancreatic volume loss was not significantly associated with the number of bevacizumab cycles. These findings suggest that bevacizumab-associated pancreatic volume reduction may involve more complex treatment-related or patient-related mechanisms beyond treatment duration alone.

The most notable clinical observation in our study was the association between treatment-emergent pancreatic volume loss and survival outcomes. In the main multivariable Cox regression models, increasing pancreatic volume loss corresponded to shorter bevacizumab-line PFS and OS. Notably, each 1% increment in pancreatic volume loss was linked to an increased risk of both progression and death, independently of other clinically relevant covariates. Importantly, established adverse prognostic factors, including poor performance status and peritoneal metastasis, also remained associated with survival outcomes, supporting the clinical validity of the models. In addition, albumin change was consistently associated with both PFS and OS, suggesting that nutritional and inflammatory deterioration during treatment may contribute to poorer outcomes. Therefore, pancreatic volume loss should not be interpreted as an isolated determinant of prognosis, but rather as a treatment-emergent imaging finding associated with survival outcomes.

The sensitivity analysis performed in patients receiving first-line bevacizumab showed that the association between pancreatic volume loss and PFS persisted in this subgroup. This finding suggests that the association observed in the main analysis was not solely attributable to differences in the bevacizumab treatment line. However, pancreatic volume loss did not retain independent significance in the corresponding OS analysis. Therefore, its prognostic value should be interpreted with caution.

The 3-month landmark analyses provided additional support for the association between pancreatic volume loss and PFS by reducing potential time-dependent measurement bias. Among patients eligible for the landmark analysis, pancreatic volume loss continued to show a significant relationship with PFS. Changes in NLR and amylase were also independently associated with both PFS and OS in the landmark analyses. In contrast, this relationship was not observed in the corresponding OS analysis; instead, ECOG performance status, peritoneal metastasis, BRAF mutation, and changes in NLR and amylase emerged as the main factors related to survival. These results indicate that pancreatic volume loss may have a more stable link with disease progression than with overall survival. The effect on OS may be more strongly shaped by subsequent treatments, disease course, and other clinical factors.

Several methodological constraints should be taken into account when evaluating these findings. The retrospective nature of the study, its reliance on a single-center experience, and the relatively modest sample size may limit the extent to which the results can be extrapolated to broader patient populations. Although previous studies did not observe comparable pancreatic volume reduction in chemotherapy-only control groups, the absence of such a control group in the present study limits the ability to definitively distinguish bevacizumab-specific effects from changes related to chemotherapy exposure or disease course. Although patients with major conditions known to affect pancreatic morphology were excluded and paired CT images were evaluated using predefined criteria, unmeasured factors and variability in imaging timing may have influenced volumetric assessment. In addition, information regarding comorbidities, primary tumor resection, and previous metastasectomy was not consistently available because of the retrospective design and therefore could not be included in the survival analyses. Therefore, these findings should be regarded as hypothesis-generating and require validation in larger prospective studies.

## 5. Conclusions

In conclusion, pancreatic volume significantly decreased during bevacizumab-based therapy in patients with metastatic colorectal cancer. Greater pancreatic volume loss was associated with shorter bevacizumab-line PFS and OS in the main analyses, while sensitivity analyses suggested a more consistent association with disease progression than with overall survival. These findings support an association between treatment-emergent pancreatic volume loss and survival outcomes, particularly disease progression. To our knowledge, this is the first study to evaluate the association between treatment-emergent pancreatic volume loss and survival outcomes in this patient population. Further prospective studies are needed to validate these findings and to clarify the clinical relevance of pancreatic volume assessment during bevacizumab-based therapy.

## Figures and Tables

**Table 1:** Baseline Characteristics of Patients Receiving Bevacizumab-Based Therapy (*N* = 105).

Variable	Overall (*N* = 105)
Age, years	68.6 (58.3–74.1)
<65 years, *n* (%)	37 (35.2)
≥65 years, *n* (%)	68 (64.8)
Sex, *n* (%)	
Male	46 (43.8)
Female	59 (56.2)
Body mass index, kg/m^2^	25.2 ± 4.6
Primary tumor location, *n* (%)	
Right-sided colon	40 (38.1)
Left-sided colon	38 (36.2)
Rectum	27 (25.7)
Stage at initial diagnosis, *n* (%)	
Stage II	5 (4.8)
Stage III	14 (13.3)
Stage IV	86 (81.9)
Metastatic sites, *n* (%)	
Liver metastasis	76 (72.4)
Lung metastasis	26 (24.8)
Peritoneal metastasis	24 (22.9)
Lymph node metastasis	25 (23.8)
Bone metastasis	6 (5.7)
Ovarian metastasis	3 (2.9)
Number of metastatic sites, *n* (%)	
1	59 (56.2)
≥2	46 (43.8)
Molecular characteristics, *n* (%)	
KRAS mutation	71 (67.6)
NRAS mutation	5 (4.8)
BRAF mutation	8 (7.6)
HER2-positive disease	5 (4.8)
MSI-H/dMMR	3 (2.9)
Treatment characteristics	
First-line bevacizumab	86 (81.9)
Second-line bevacizumab	19 (18.1)
Chemotherapy backbone, *n* (%)	
FOLFOX + bevacizumab	72 (68.6)
FOLFIRI + bevacizumab	30 (28.6)
Capecitabine + bevacizumab	3 (2.9)
Number of bevacizumab cycles	12 (7–20)
Baseline laboratory parameters	
Hemoglobin, g/dL	11.7 ± 1.9
Platelet count, ×10^9^/L	359.7 ± 122.0
Albumin, g/L	38.8 ± 4.3
Amylase, U/L	64.2 ± 34.4
NLR	3.4 (2.4–4.7)
Baseline pancreatic volume, mL	89.9 ± 6.1

Abbreviations: BMI, body mass index; BRAF, v-Raf murine sarcoma viral oncogene homolog B1; dMMR, deficient mismatch repair; FOLFIRI, fluorouracil, leucovorin, and irinotecan; FOLFOX, fluorouracil, leucovorin, and oxaliplatin; HER2, human epidermal growth factor receptor 2; KRAS, Kirsten rat sarcoma viral oncogene homolog; MSI-H, microsatellite instability-high; NLR, neutrophil-to-lymphocyte ratio; NRAS, neuroblastoma RAS viral oncogene homolog; SD, standard deviation. Values are presented as *n* (%), mean ± standard deviation, or median (interquartile range), as appropriate.

**Table 2:** Changes in Pancreatic Volume and Laboratory Parameters During Bevacizumab-Based Therapy.

Variable	Pre-Treatment	Post-Treatment	*p* Value
Pancreatic volume, mL	89.9 ± 6.1	83.6 ± 5.1	<0.001
Albumin, g/L	38.8 ± 4.3	35.7 ± 6.6	<0.001
Amylase, U/L	54.0 (43.0–77.0)	50.0 (34.0–81.0)	0.518
Hemoglobin, g/dL	11.7 ± 1.9	11.6 ± 2.0	0.551
Platelet count, ×10^9^/L	359.7 ± 122.0	237.0 ± 106.8	<0.001
NLR	3.4 (2.4–4.7)	3.4 (1.9–5.4)	0.889

Continuous variables are expressed as mean ± standard deviation or median (interquartile range), according to their distribution. Pre- and post-treatment measurements were compared with the paired *t*-test or Wilcoxon signed-rank test, as appropriate for data distribution. Abbreviations: NLR, neutrophil-to-lymphocyte ratio; SD, standard deviation.

**Table 3:** Factors Associated With Percentage Pancreatic Volume Loss.

Variable	Spearman r	*p* Value
ΔNLR (%)	−0.013	0.895
ΔAmylase (%)	−0.199	0.042
ΔAlbumin (%)	−0.014	0.889
ΔPlatelet (%)	−0.013	0.892
Number of bevacizumab cycles	−0.119	0.225
Age, years	0.102	0.300
BMI, kg/m^2^	−0.055	0.579

Abbreviations: BMI, body mass index; NLR, neutrophil-to-lymphocyte ratio. Spearman correlation analysis was performed using percentage pancreatic volume loss as the dependent variable.

**Table 4:** Univariable and Multivariable Linear Regression Analyses for Factors Associated With Percentage Pancreatic Volume Loss.

Variable	Univariable β (95% CI)	*p* Value	Multivariable β (95% CI)	*p* Value
Age, years	0.015 (−0.031 to 0.062)	0.514	0.025 (−0.026 to 0.075)	0.337
BMI, kg/m^2^	−0.025 (−0.152 to 0.102)	0.697	−0.040 (−0.178 to 0.099)	0.571
Number of bevacizumab cycles	−0.045 (−0.105 to 0.014)	0.135	−0.044 (−0.106 to 0.018)	0.164
ΔNLR (%)	−0.002 (−0.006 to 0.003)	0.496	−0.003 (−0.008 to 0.002)	0.198
ΔAlbumin (%)	0.004 (−0.004 to 0.011)	0.345	0.005 (−0.003 to 0.012)	0.216
ΔAmylase (%)	−0.006 (−0.014 to 0.002)	0.138	−0.006 (−0.014 to 0.002)	0.139
ΔPlatelet (%)	0.002 (−0.009 to 0.013)	0.710	0.004 (−0.007 to 0.016)	0.467

Dependent variable: Percentage pancreatic volume loss. Abbreviations: BMI, body mass index; NLR, neutrophil-to-lymphocyte ratio; CI, confidence interval.

**Table 5:** Univariable and Multivariable Cox Regression Analyses for Bevacizumab-Line Progression-Free Survival.

Variable	Univariable HR (95% CI)	*p* Value	Multivariable HR (95% CI)	*p* Value
Age ≥ 65 years	0.96 (0.62–1.48)	0.851	—	—
Male sex	1.42 (0.94–2.16)	0.096	1.09 (0.70–1.69)	0.701
BMI, kg/m^2^	0.99 (0.94–1.04)	0.656	—	—
ECOG ≥ 2	1.91 (1.17–3.13)	0.010	1.78 (1.06–2.99)	0.028
Right-sided primary	0.97 (0.63–1.48)	0.879	—	—
Liver metastasis	1.47 (0.91–2.39)	0.118	—	—
Lung metastasis	0.76 (0.47–1.24)	0.277	—	—
Peritoneal metastasis	1.62 (1.00–2.63)	0.052	2.06 (1.25–3.39)	0.005
≥2 metastatic sites	1.09 (0.72–1.65)	0.693	—	—
KRAS mutation	0.91 (0.58–1.41)	0.664	—	—
BRAF mutation	1.51 (0.69–3.27)	0.300	—	—
Second-line bevacizumab	1.17 (0.69–1.98)	0.567	—	—
Irinotecan-based backbone	1.17 (0.74–1.85)	0.491	—	—
Stage IV at diagnosis	1.26 (0.71–2.23)	0.435	—	—
Pancreatic volume loss (%)	1.09 (1.05–1.13)	0.002	1.06 (1.02–1.10)	0.032
ΔNLR (%)	1.003 (1.001–1.004)	<0.001	1.002 (1.000–1.004)	0.093
ΔAlbumin (%)	0.98 (0.97–0.99)	<0.001	0.99 (0.97–1.00)	0.015
ΔAmylase (%)	0.995 (0.991–0.998)	0.005	0.997 (0.993–1.000)	0.086
ΔPlatelet (%)	1.005 (1.001–1.009)	0.019	1.002 (0.998–1.007)	0.286

Results are expressed as hazard ratios (HRs) with 95% confidence intervals (CIs). Bevacizumab-line progression-free survival was defined as the interval from the start of bevacizumab-containing therapy to disease progression or death from any cause; the earliest of these events was considered the PFS endpoint. Patients without progression at the last assessment were censored. PFS events were recorded in 92 of 105 patients (87.6%). Variables showing *p* < 0.10 in univariable analysis, together with clinically relevant covariates, were selected as candidate variables for the multivariable model. For continuous variables, HRs represent the effect of each 1-unit increase, whereas for percentage-change parameters, HRs correspond to each 1% increase. Delta (Δ) values were calculated using the formula [(post-treatment value − pre-treatment value)/pre-treatment value] × 100. Collinearity among variables entered into the multivariable models was checked using variance inflation factor values, and all VIF values were <5. Chemotherapy backbone was grouped as irinotecan-containing or non-irinotecan-containing regimens, with non-irinotecan-containing regimens used as the reference category. Because chemotherapy backbone was not significant in univariable analysis, it was not included in the primary multivariable models. Stage at initial diagnosis was analyzed as stage IV versus stage II–III because only a small number of patients had stage II or III disease at diagnosis. Abbreviations: BMI, body mass index; CI, confidence interval; ECOG, Eastern Cooperative Oncology Group; HR, hazard ratio; KRAS, Kirsten rat sarcoma viral oncogene homolog; NLR, neutrophil-to-lymphocyte ratio; PFS, progression-free survival; VIF, variance inflation factor.

**Table 6:** Univariable and Multivariable Cox Regression Analyses for Bevacizumab-Line Overall Survival.

Variable	Univariable HR (95% CI)	*p* Value	Multivariable HR (95% CI)	*p* Value
Age ≥ 65 years	1.12 (0.69–1.84)	0.649	—	—
Male sex	1.38 (0.87–2.21)	0.175	—	—
BMI, kg/m^2^	0.97 (0.92–1.03)	0.365	—	—
ECOG ≥ 2	2.92 (1.73–4.93)	<0.001	2.98 (1.71–5.22)	<0.001
Right-sided primary	1.07 (0.66–1.73)	0.782	—	—
Liver metastasis	1.41 (0.81–2.46)	0.230	—	—
Lung metastasis	0.83 (0.48–1.44)	0.513	—	—
Peritoneal metastasis	1.80 (1.06–3.06)	0.030	2.60 (1.49–4.55)	<0.001
≥2 metastatic sites	1.24 (0.78–1.98)	0.369	—	—
KRAS mutation	1.01 (0.60–1.69)	0.970	—	—
BRAF mutation	2.36 (1.07–5.18)	0.033	3.19 (0.93–8.43)	0.103
Second-line bevacizumab	1.28 (0.71–2.30)	0.418	—	—
Irinotecan-based backbone	1.31 (0.78–2.20)	0.302	—	—
Stage IV at diagnosis	1.13 (0.59–2.16)	0.708	—	—
Pancreatic volume loss (%)	1.10 (1.01–1.21)	0.029	1.08 (1.01–1.15)	0.041
ΔNLR (%)	1.003 (1.001–1.004)	0.001	1.001 (1.000–1.003)	0.135
ΔAlbumin (%)	0.98 (0.96–0.99)	<0.001	0.98 (0.96–0.99)	0.002
ΔAmylase (%)	0.993 (0.988–0.998)	0.002	0.996 (0.990–1.002)	0.062
ΔPlatelet (%)	1.003 (0.999–1.007)	0.114	—	—

Results are expressed as hazard ratios (HRs) with 95% confidence intervals (CIs). Bevacizumab-line overall survival was defined as the interval from the start of bevacizumab-containing therapy to death from any cause. Patients who were alive at the last follow-up were censored in the OS analysis. Death events were recorded in 71 of 105 patients (67.6%). Variables with *p* < 0.10 in univariable analysis, together with clinically relevant covariates, were selected as candidate variables for the multivariable model. For continuous variables, HRs represent the effect of each 1-unit increase, whereas for percentage-change parameters, HRs correspond to each 1% increase. Delta (Δ) values were calculated using the formula [(post-treatment value − pre-treatment value)/pre-treatment value] × 100. Collinearity among variables entered into the multivariable models was checked using variance inflation factor values, and all VIF values were <5. Stage at initial diagnosis was analyzed as stage IV versus stage II–III because only a small number of patients had stage II or III disease at diagnosis. Chemotherapy backbone was grouped as irinotecan-containing or non-irinotecan-containing regimens, with non-irinotecan-containing regimens used as the reference category. Because chemotherapy backbone was not significant in univariable analysis, it was not included in the primary multivariable models. Abbreviations: BMI, body mass index; BRAF, v-Raf murine sarcoma viral oncogene homolog B1; CI, confidence interval; ECOG, Eastern Cooperative Oncology Group; HR, hazard ratio; KRAS, Kirsten rat sarcoma viral oncogene homolog; NLR, neutrophil-to-lymphocyte ratio; OS, overall survival; VIF, variance inflation factor.

## Data Availability

The datasets generated and/or analyzed during the current study are not publicly available due to institutional and patient privacy regulations but are available from the corresponding author upon reasonable request.

## Supplementary Materials

The following supporting information can be downloaded at https://www.mdpi.com/article/10.3390/medicina62081459/s1: Supplementary Table S1. Survival Outcomes in the Overall Cohort and First-Line Bevacizumab Subgroup; Supplementary Table S2. Sensitivity Cox Regression Analysis for Bevacizumab-Line Progression-Free Survival in Patients Receiving First-Line Bevacizumab; Supplementary Table S3. Sensitivity Cox Regression Analysis for Bevacizumab-Line Overall Survival in Patients Receiving First-Line Bevacizumab; Supplementary Table S4. Fixed 3-Month Landmark Cox Regression Analysis for Bevacizumab-Line Progression-Free Survival; Supplementary Table S5. Fixed 3-Month Landmark Cox Regression Analysis for Bevacizumab-Line Overall Survival; Supplementary Figure S1. Restricted cubic spline analysis of the non-linear relationship between pancreatic volume loss and survival outcomes. (A) Bevacizumab-line Progression-Free Survival (PFS). (B) Bevacizumab-line Overall Survival (OS).
