# Supplementary material for: Association Between Treatment-Emergent Pancreatic Volume Loss and Survival Outcomes in Patients with Metastatic Colorectal Cancer Receiving Bevacizumab
Source: Medicina (Kaunas). 2026 Jul 28;62(8):1459. doi: 10.3390/medicina62081459 (PMC13514549; doi:10.3390/medicina62081459)
Supplement: Supplementary file 1 [file medicina-62-01459-s001.zip › medicina-4418170-supplementary.pdf]

Supplementary Table S1. Survival Outcomes in the Overall Cohort and First-Line Bevacizumab Subgroup

Survival Outcomes

Outcome	N	Events, n (%)	Censored, n (%)	Median survival, months (95% CI)
Bevacizumab-line PFS	105	92 (87.6)	13 (12.4)	8.84 (6.77–11.04)
Bevacizumab-line OS	105	71 (67.6)	34 (32.4)	14.23 (10.87–18.79)
Overall OS from diagnosis	105	71 (67.6)	34 (32.4)	20.86 (17.15–25.72)
First-line PFS	105	94 (89.5)	11 (10.5)	11.93 (10.45–12.81)

Sensitivity analysis restricted to first-line bevacizumab recipients

Outcome	N	Events, n (%)	Censored, n (%)	Median survival, months (95% CI)
Bevacizumab-line PFS	86	75 (87.2)	11 (12.8)	8.84 (6.80–11.37)
Bevacizumab-line OS	86	57 (66.3)	29 (33.7)	15.97 (10.87–19.45)
Overall OS from diagnosis	86	57 (66.3)	29 (33.7)	19.42 (14.62–29.17)
First-line PFS	86	75 (87.2)	11 (12.8)	11.99 (10.45–13.60)

Median survival estimates and corresponding 95% confidence intervals were calculated using the Kaplan–Meier method. Bevacizumab-line PFS and OS were calculated from the initiation of bevacizumab-based therapy. First-line PFS was calculated from the initiation of first-line systemic therapy. Overall OS from diagnosis was calculated from the date of diagnosis to death from any cause. Patients without an event at the last follow-up were censored.

Abbreviations: CI, confidence interval; OS, overall survival; PFS, progression-free survival.

Supplementary Table S2. Sensitivity Cox Regression Analysis for Bevacizumab-Line Progression-Free Survival in Patients Receiving First-Line Bevacizumab

Variable	Univariable HR (95% CI)	P value	Multivariable HR (95% CI)	P value
Age ≥65 years	0.97 (0.60–1.59)	0.911	—	—
Male sex	1.29 (0.81–2.04)	0.283	—	—
BMI, kg/m²	0.98 (0.93–1.04)	0.490	—	—
ECOG ≥2	1.83 (1.08–3.10)	0.025	1.70 (0.97–2.96)	0.062
Right-sided primary	0.99 (0.62–1.58)	0.968	—	—
Liver metastasis	1.59 (0.94–2.70)	0.084	1.53 (0.89–2.62)	0.123
Lung metastasis	0.74 (0.44–1.27)	0.278	—	—
Peritoneal metastasis	1.47 (0.87–2.49)	0.153	—	—
≥2 metastatic sites	0.92 (0.58–1.47)	0.738	—	—
KRAS mutation	0.92 (0.55–1.55)	0.764	—	—
BRAF mutation	1.62 (0.74–3.55)	0.228	—	—
Irinotecan-based backbone	1.28 (0.65–2.49)	0.476	—	—
Pancreatic volume loss (%)	1.07 (1.02–1.12)	0.029	1.04 (1.01–1.07)	0.043
ΔNLR (%)	1.002 (1.000–1.004)	0.013	1.001 (0.999–1.003)	0.472
ΔAlbumin (%)	0.981 (0.969–0.993)	0.002	0.985 (0.971–0.998)	0.029
ΔAmylase (%)	0.996 (0.992–1.000)	0.035	0.998 (0.994–1.002)	0.259
ΔPlatelet (%)	1.005 (0.999–1.010)	0.087	1.003 (0.997–1.009)	0.313

Values are presented as hazard ratios (HRs) with 95% confidence intervals (CIs). Bevacizumab-line progression-free survival was calculated from the initiation of bevacizumab-based therapy to disease progression or death from any cause, whichever occurred first. Patients without progression at the last follow-up were censored. A total of 75 progression-free survival events occurred among 86 patients (87.2%). Variables with a P value <0.10 in univariable analysis and clinically relevant covariates were considered for inclusion in the multivariable model. For continuous variables, HRs indicate the effect per 1-unit increase; for percentage-change variables, HRs indicate the effect per 1% increase. Delta (Δ) values were calculated as [(post-treatment value − pre-treatment value) / pre-treatment value] × 100. Chemotherapy backbone was categorized as irinotecan-based versus non-irinotecan-based therapy. Multicollinearity was assessed using variance inflation factors, and all included variables had VIF values <5.

Abbreviations: BMI, body mass index; CI, confidence interval; ECOG, Eastern Cooperative Oncology Group; HR, hazard ratio; KRAS, Kirsten rat sarcoma viral oncogene homolog; NLR, neutrophil-to-lymphocyte ratio; PFS, progression-free survival; VIF, variance inflation factor.

Supplementary Table S3. Sensitivity Cox Regression Analysis for Bevacizumab-Line Overall Survival in Patients Receiving First-Line Bevacizumab

Variable	Univariable HR (95% CI)	P value	Multivariable HR (95% CI)	P value
Age ≥65 years	1.51 (0.83–2.73)	0.176	—	—
Male sex	1.36 (0.81–2.29)	0.245	—	—
BMI, kg/m²	0.98 (0.92–1.04)	0.495	—	—
ECOG ≥2	2.86 (1.62–5.05)	<0.001	3.31 (1.78–6.18)	<0.001
Right-sided primary	1.10 (0.64–1.87)	0.737	—	—
Liver metastasis	1.30 (0.72–2.35)	0.379	—	—
Lung metastasis	0.94 (0.53–1.68)	0.837	—	—
Peritoneal metastasis	1.65 (0.92–2.95)	0.091	2.43 (1.31–4.52)	0.005
≥2 metastatic sites	1.14 (0.68–1.92)	0.623	—	—
KRAS mutation	0.98 (0.54–1.78)	0.953	—	—
BRAF mutation	2.44 (1.09–5.42)	0.029	4.39 (1.80–10.70)	0.001
Irinotecan-based backbone	1.40 (0.66–2.98)	0.381	—	—
Pancreatic volume loss (%)	1.11 (1.01–1.22)	0.033	1.06 (0.97–1.17)	0.196
ΔNLR (%)	1.002 (1.000–1.004)	0.029	1.001 (0.998–1.003)	0.623
ΔAlbumin (%)	0.977 (0.963–0.991)	0.002	0.975 (0.959–0.990)	0.002
ΔAmylase (%)	0.994 (0.989–0.999)	0.017	0.996 (0.992–1.001)	0.098
ΔPlatelet (%)	1.002 (0.998–1.007)	0.318	—	—

Values are presented as hazard ratios (HRs) with 95% confidence intervals (CIs). Bevacizumab-line overall survival was calculated from the initiation of bevacizumab-based therapy to death from any cause. Patients alive at the last follow-up were censored. A total of 57 deaths occurred among 86 patients (66.3%). Variables with a P value <0.10 in univariable analysis and clinically relevant covariates were considered for inclusion in the multivariable model. For continuous variables, HRs indicate the effect per 1-unit increase; for percentage-change variables, HRs indicate the effect per 1% increase. Delta (Δ) values were calculated as [(post-treatment value − pre-treatment value) / pre-treatment value] × 100. Chemotherapy backbone was categorized as irinotecan-based versus non-irinotecan-based therapy. Multicollinearity was assessed using variance inflation factors, and all included variables had VIF values <5.

Abbreviations: BMI, body mass index; BRAF, v-Raf murine sarcoma viral oncogene homolog B1; CI, confidence interval; ECOG, Eastern Cooperative Oncology Group; HR, hazard ratio; KRAS, Kirsten rat sarcoma viral oncogene homolog; NLR, neutrophil-to-lymphocyte ratio; OS, overall survival; VIF, variance inflation factor.

Supplementary Table S4. Fixed 3-Month Landmark Cox Regression Analysis for Bevacizumab-Line Progression-Free Survival

Variable	Univariable HR (95% CI)	P value	Multivariable HR (95% CI)	P value
ECOG ≥2	1.86 (1.10–3.16)	0.020	1.46 (0.85–2.53)	0.172
Liver metastasis	1.73 (1.02–2.93)	0.042	1.80 (0.98–3.09)	0.062
Pancreatic volume loss (%)	1.09 (1.01–1.19)	0.023	1.08 (1.01–1.15)	0.047
ΔNLR (%)	1.003 (1.001–1.005)	<0.001	1.002 (1.001–1.004)	0.006
ΔAmylase (%)	0.995 (0.991–0.999)	0.010	0.995 (0.991–0.999)	0.011

Values are presented as hazard ratios (HRs) with 95% confidence intervals (CIs). A fixed 3-month landmark sensitivity analysis was performed to reduce potential time-dependent measurement bias related to treatment-emergent variables. Patients who experienced disease progression, died, or were censored before the 3-month landmark were excluded from this analysis. Survival time was recalculated from the 3-month landmark. In this landmark analysis, 84 progression-free survival events occurred among 97 eligible patients. Variables with a P value <0.10 in univariable analysis and clinically relevant covariates were considered for inclusion in the multivariable model. For continuous variables, HRs indicate the effect per 1-unit increase; for percentage-change variables, HRs indicate the effect per 1% increase. Delta (Δ) values were calculated as [(post-treatment value − pre-treatment value) / pre-treatment value] × 100. Percentage pancreatic volume loss was calculated as [(pre-treatment pancreatic volume − post-treatment pancreatic volume) / pre-treatment pancreatic volume] × 100. Multicollinearity was assessed using variance inflation factors, and all included variables had VIF values <5.

Abbreviations: CI, confidence interval; ECOG, Eastern Cooperative Oncology Group; HR, hazard ratio; NLR, neutrophil-to-lymphocyte ratio; PFS, progression-free survival; VIF, variance inflation factor.

Supplementary Table S5. Fixed 3-Month Landmark Cox Regression Analysis for Bevacizumab-Line Overall Survival

Variable	Univariable HR (95% CI)	P value	Multivariable HR (95% CI)	P value
ECOG ≥2	2.67 (1.55–4.59)	<0.001	2.56 (1.42–4.59)	0.002
Peritoneal metastasis	1.76 (1.02–3.02)	0.041	2.42 (1.37–4.28)	0.002
BRAF mutation	2.49 (1.13–5.47)	0.024	2.70 (1.17–6.27)	0.021
Pancreatic volume loss (%)	1.11 (1.02–1.22)	0.021	1.05 (0.97–1.15)	0.236
ΔNLR (%)	1.003 (1.001–1.004)	<0.001	1.002 (1.001–1.004)	0.005
ΔAmylase (%)	0.993 (0.988–0.997)	0.003	0.994 (0.989–0.998)	0.009

Values are presented as hazard ratios (HRs) with 95% confidence intervals (CIs). A fixed 3-month landmark sensitivity analysis was performed to reduce potential time-dependent measurement bias related to treatment-emergent variables. Patients who died or were censored before the 3-month landmark were excluded from this analysis. Survival time was recalculated from the 3-month landmark. In this landmark analysis, 69 deaths occurred among 103 eligible patients. Variables with a P value <0.10 in univariable analysis and clinically relevant covariates were considered for inclusion in the multivariable model. For continuous variables, HRs indicate the effect per 1-unit increase; for percentage-change variables, HRs indicate the effect per 1% increase. Delta (Δ) values were calculated as [(post-treatment value − pre-treatment value) / pre-treatment value] × 100. Percentage pancreatic volume loss was calculated as [(pre-treatment pancreatic volume − post-treatment pancreatic volume) / pre-treatment pancreatic volume] × 100. Multicollinearity was assessed using variance inflation factors, and all included variables had VIF values <5.

Abbreviations: BRAF, v-Raf murine sarcoma viral oncogene homolog B1; CI, confidence interval; ECOG, Eastern Cooperative Oncology Group; HR, hazard ratio; NLR, neutrophil-to-lymphocyte ratio; OS, overall survival; VIF, variance inflation factor.

Supplementary Figure S1. Restricted cubic spline analysis of the non-linear relationship between pancreatic volume loss and survival outcomes. (A) Bevacizumab-line Progression-Free Survival (PFS). (B) Bevacizumab-line Overall Survival (OS)

(A)

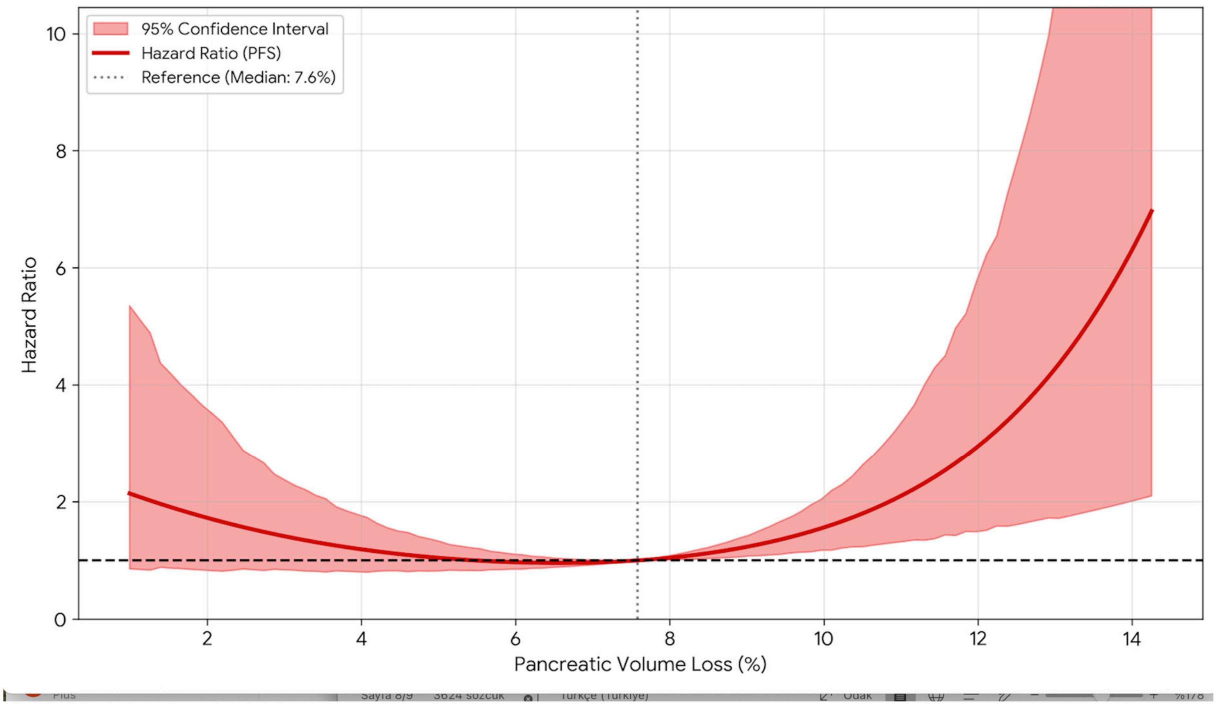

(B)

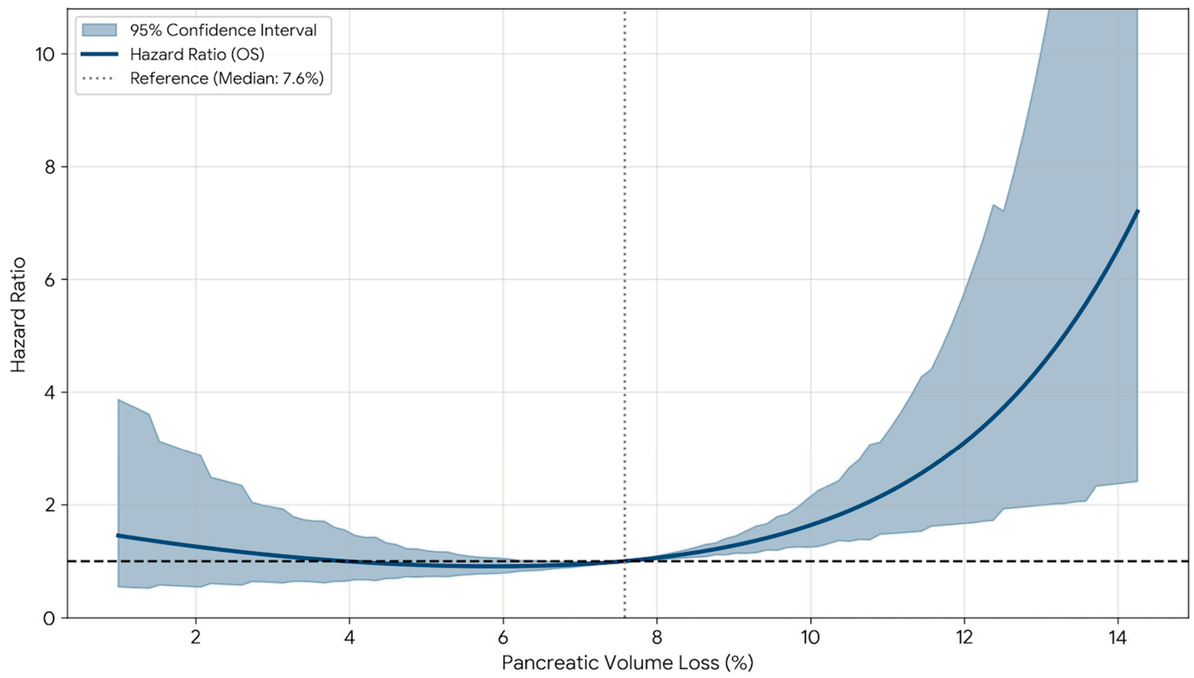

Abbreviations: CI, confidence interval; HR, hazard ratio; OS, overall survival; PFS, progression-free survival; RCS, restricted cubic spline.
